# Quality Improvement Identifies Healthcare Transition Disparities in Adolescents with Congenital Heart Disease and Disabilities

**DOI:** 10.1097/pq9.0000000000000732

**Published:** 2024-05-27

**Authors:** Catherine C. Allen, Briana L. Swanson, Xiao Zhang, Ryan J. Coller, Krisjon R. Olson

**Affiliations:** * From the Division of Pediatric Cardiology, Department of Pediatrics, University of Wisconsin School of Medicine and Public Health, Madison, Wisc.; † Division of Cardiovascular Medicine, Department of Medicine, University of Wisconsin School of Medicine and Public Health, Madison, Wisc.; ‡ Division of Pediatric Hospital Medicine, Department of Pediatrics, University of Wisconsin School of Medicine and Public Health, Madison, Wisc.

## Abstract

**Introduction:**

We aim to implement healthcare transition (HCT) education for youth with congenital heart disease (CHD) and assess HCT preparedness for cardiac self-care.

**Methods:**

An HCT clinic was implemented at an academic pediatric cardiology clinic for CHD youth 17 years of age and older. An educator used transition readiness assessment questionnaires and discussed HCT material. The percentage of eligible youth who received HCT education and the cause for missed occurrences were tracked. Plan-do-study-act cycles began in August 2020 to improve the number of youths reached. Secondary analyses assessed improvement differences among those without cardiac procedures or disabilities.

**Results:**

HCT education provision improved from a mean of 38% to 73% in the 17-year and older age group by December 2022. Communication failure was the leading cause of missed visits in 2021 (30%), reduced to 0 by 2022 following plan-do-study-act cycles. Other missed HCT visits included clinic add-ons after screening, limited staff availability, and unidentified eligibility. Readiness assessments were similar for youth with and without prior cardiac procedures, for example, confidence in taking charge of their health care (*P* = 0.47) and moving to adult care (*P* = 0.22). Adolescents with disabilities were significantly less confident than those without disabilities in taking charge of their heart health care (6.3 versus 7.5, *P* = 0.04) and moving to adult care (4.9 versus 7.4, *P* < 0.001).

**Conclusions:**

Implementation of a CHD HCT clinic improved successful education delivery. Provider engagement and clinic staffing are important for sustainability. HCT knowledge gaps exist for all adolescents, yet those with disabilities had the greatest deficits.

## INTRODUCTION

Healthcare transition (HCT) plays a crucial role in encouraging youth to be active in managing their own healthcare needs throughout their lives. HCT is recommended to =occur over a continuum for all youth beginning at 12 years of age and should include preparation, education, and services needed to transition successfully to adult healthcare systems.^1–4^ Disability-related needs should be addressed within HCT^5^; however, data from the National Survey of Children’s Health suggest 83% of youth with special healthcare needs do not meet national HCT standards.^6^ Although the optimal methods to provide HCT have not yet been established, electronic medical records (EMRs) and patient navigators have been utilized to improve HCT.^7,8^ The one in 100 children born with congenital heart disease (CHD) is a particularly important HCT population. Advancements in therapies have dramatically extended their life expectancy, and there are now more adults living with CHD than children.^9–11^ Despite these improvements, as many as 50%–75% are lost to follow up in the transition from pediatric to adult congenital cardiac care.^12,13^

The American Heart Association,^14^ American Academy of Pediatrics,^15^ and the Centers for Disease Control and Prevention^16^ recommend patients with CHD continue specialized lifelong care to decrease the known risks of poorly coordinated transition to adult care, for example, progression of preventable illness, anxiety, intensive care admission, or premature death.^17–22^ However, many youths with CHD are not getting the information and support needed to make a successful transition.^17,23–25^ The importance of HCT and the absence of effective evidence-based HCT tools underscore the urgency to identify factors impacting HCT.

In our center, we identified a need for a robust adolescent HCT process to reach youth with CHD more uniformly and prepare them for transitioning to adult congenital cardiology. The specific, measurable, achievable, relevant, time-based (SMART) aim was to offer adolescent HCT education at our primary clinic site, with each routine pediatric cardiology clinic visit to at least 75% of patients with CHD 17 years and older by January 2022. A longer-term global aim is to reach at least 95% of those 12 years and older. A secondary aim was to utilize a standardized transition readiness assessment (TRA) questionnaire to assess the youth’s HCT confidence and preparation. We hypothesized that those with previous cardiac surgeries or catheterizations, as well as those with disabilities, would have lower confidence and knowledge due to the neurodevelopmental impact on cardiopulmonary bypass (CPB)^26^ or added HCT challenges with disabilities.^25,27–29^ If confirmed, a need to develop additional processes for those more vulnerable populations is anticipated.

## METHODS

### Context

The CHD HCT clinic is located in the pediatric cardiology division of a tertiary academic medical center. Approximately 250 adolescents with CHD 12 years of age and older are seen at least annually, approximately 35% of those 17 years of age and older. Patients with CHD transition care to the same institution’s adult congenital heart disease (ACHD) clinic. The pediatric cardiology and ACHD primary clinic locations are on the same campus. The medical center uses Epic EMR (Epic Systems, Verona, Wisc.). Additional regional outreach clinics were not included to limit multisite variables. In 2018, a pediatric-to-adult congenital cardiology HCT initiative was started, consisting of an educational folder with handouts highlighting similarities and differences between pediatric and adult practices, general healthcare milestones according to age, and ACHD clinic information. Initially, the intent was for pediatric cardiologists and medical assistants to distribute the folders during cardiology clinic visits. However, this process was unreliable due to staffing changes and inconsistency in identifying relevant patients.

### Interventions

**Fig. 1.**
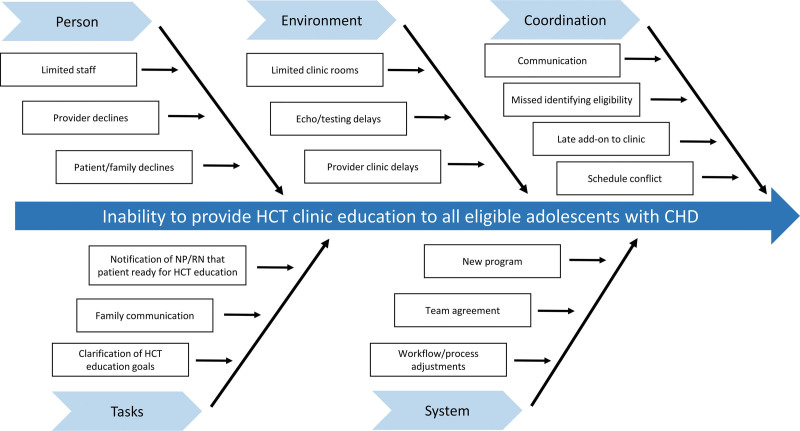
Fishbone diagram. NP, nurse practitioner; RN, registered nurse.

**Fig. 2.**
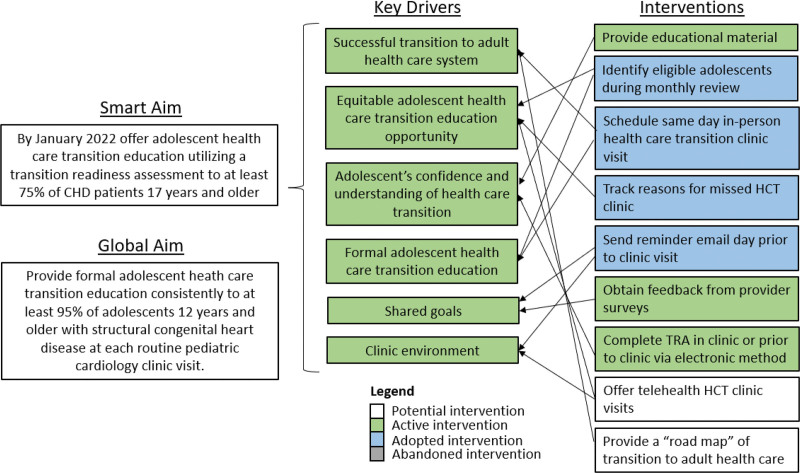
Key driver diagram.

**Fig. 3.**
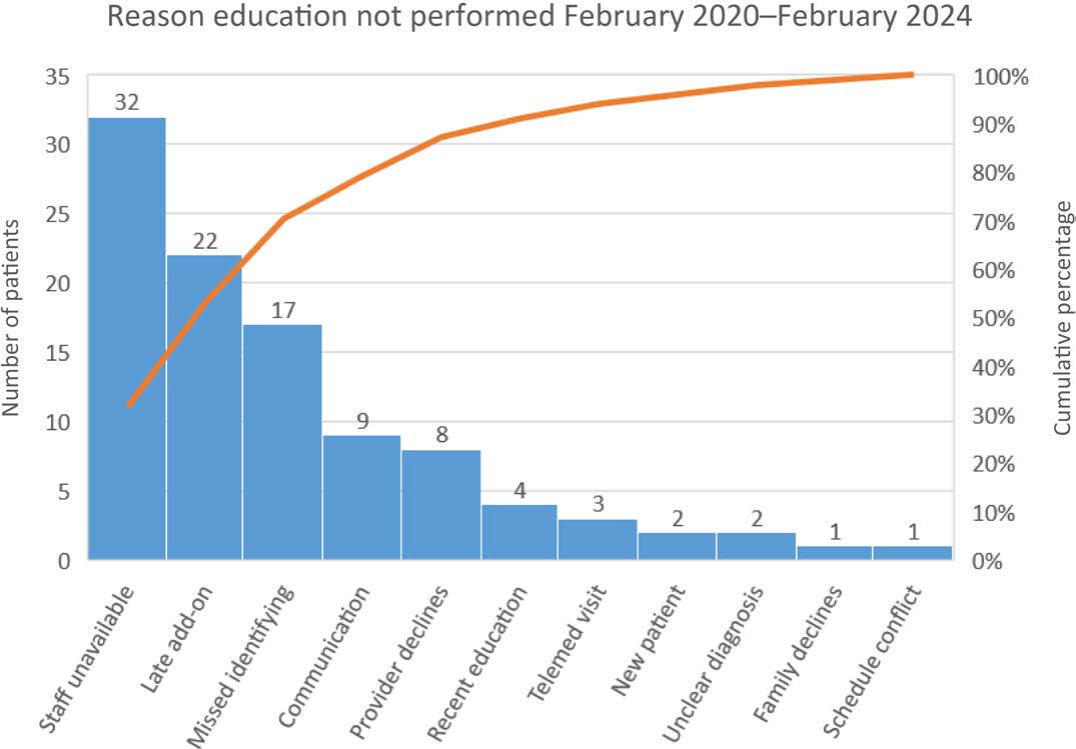
Pareto chart indicating the reason that the healthcare transition clinic was not completed in 17-year-old and older adolescents with congenital heart disease from February 2020 to February 2024.

An HCT clinic was established in February 2020 to overcome inconsistencies within the initial approach. The clinic combined an in-person visit focused on HCT education and the use of a TRA questionnaire to guide educational topics.^2^ The initial CHD HCT clinic team consisted of champion representatives, including a pediatric cardiology physician, an adult congenital cardiology nurse practitioner, and a registered nurse. The HCT team expanded also to include a research scientist, medical anthropologist, and HCT physician to assist with scientific and quality evaluation. The HCT team created fishbone (Fig. 1) and key driver (Fig. 2) diagrams to identify improvement areas and inform strategies used in serial plan-do-study-act (PDSA) cycles. A survey of the pediatric cardiology providers was performed in 2020, 2021, and 2023 to evaluate and gain input regarding the HCT clinic, assess provider agreement in the improvement process, and receive formal feedback. Each month, reasons for missed HCT clinic visits were tracked and evaluated utilizing a Pareto chart (Fig. 3), and those findings informed additional improvement opportunities.

Adolescents eligible for the HCT clinic were those with structural CHD, including bicuspid aortic valve or other valvular disease with mild or greater stenosis or regurgitation and connective tissue disorders, 17 years or older, and scheduled for in-person cardiology clinic visits. Cardiac arrhythmias without a structural CHD indication and heart transplantation were not eligible as other HCT processes are in place. Visits were initially only offered to those 17 years and older due to staffing and clinic capacity limitations. Each month, one HCT team member reviewed upcoming pediatric cardiology visits to identify eligible patients and schedule an HCT visit immediately following the upcoming cardiology clinic visit. On the day of the visit, if an HCT team member was available, the adolescent completed the TRA, followed by HCT education. If no HCT team member was available, HCT education was provided at the discretion of the cardiologist’s routine practice. The TRA examined CHD youths’ level of confidence in taking charge of their health care and in transitioning to adult-focused care, as well as knowledge of their heart diagnoses and navigating health care. After completing the TRA, a healthcare team member (nurse practitioner or registered nurse) reviewed the TRA responses to guide age-appropriate HCT-related education, for example, reviewing their cardiac diagnosis, legal implications of turning 18, carrying a medical summary, and needing lifelong congenital cardiology care.

As in-person clinics were beginning again after initial COVID-19 shutdowns, PDSA tests of change were started in August 2020, which consisted of an education session with the pediatric cardiology team (physicians, advanced practice providers, nurses) to discuss HCT clinic goals, provider concerns, collect input, and apply outcomes from that session to improve the number of youths reached. In December 2020, email notifications were sent to providers the day before the clinic visit to remind them of the HCT education as another PDSA cycle to improve communication and participation. The next PDSA cycle in April 2021 included nursing support to provide HCT education (nursing support for the HCT clinic was decreased in February 2022 due to shifting clinical roles, re-established in December 2022, and again decreased in August 2023). In addition, an HCT clinic appointment was scheduled in the EMR starting in June 2021 to be a visibly scheduled appointment to remind all team members of the HCT visit and not rely solely on email reminders.

Seventeen months after implementation (about 1 year after our clinics began fully running again after COVID-19 shutdowns), we noted successful trends in the ability to see the 17-year and older youth and provider requests to expand eligibility. Therefore, an additional PDSA test of change to include those 12–16 years of age began. In this PDSA cycle, two pediatric cardiologists who had expressed strong interest in the HCT clinic were identified and included all their 12-year-old and older CHD youth. The goals of this PDSA cycle were to gain exposure in specific cardiologists’ clinics and test the feasibility of expansion of the HCT clinic. Additional PDSA cycles were completed to continue to gain support and consistent implementation from all the pediatric cardiology providers.

### Measures

The primary outcome measure was the percentage of adolescents 17 and older receiving HCT education. Data were collected by chart review monthly to determine if the adolescent was eligible for HCT education if HCT education was completed, and if not completed, the reason documented. The percentage receiving HCT education was calculated by dividing the total number of HCT education encounters by the total number of possible HCT education encounters. We also tracked the percentage of CHD adolescents 12 years and older receiving HCT education to know baseline performance for the full scope of the population we ultimately want to reach for the longer-term global aim. Secondary outcomes, such as the youth’s confidence in and readiness for HCT, were assessed using the TRA questionnaire.^2^ Specifically, the TRA measures a youth’s confidence in taking charge of heart health care and moving to adult-focused heart care (scale ranging from 0 to 10, with ten being most confident) and knowledge of heart health and using health care (yes/no/not applicable). Process measures included reasons for missed HCT education and cardiology provider satisfaction surveys. Adolescent characteristics abstracted from the EMR included sex, age at TRA completion, CHD diagnosis, number of cardiac procedures, including surgeries and hemodynamic or interventional catheterizations, age at initial surgery with CPB, number of surgeries requiring CPB, and presence of a disability defined by documentation in the EMR by the word “disability” in a clinic note, visual, hearing, or physical impairments, or existence of an individualized education plan. Utilizing the EMR search function allowed a search of every note from any provider (ie, cardiology, primary care, neurodevelopmental providers) to broaden potential documentation indicating a disability.

### Statistical Analysis

Descriptive statistics were calculated on demographics, medical history, and responses to the TRA questionnaire. *T* tests for continuous and categorical variables were performed to compare by prior history of cardiac surgery and disability status. All analyses were performed with the statistical software STATA/SE 16.1 (StataCorp, College Station, Tex.). A *P* value of 0.05 was defined as significant. Statistical process control p-charts were used to monitor changes in the percentage of adolescents receiving HCT education using established rules for identifying special cause variation.^30^ We considered eight consecutive points above or below the centerline and any points outside the control limits to represent special cause variation, suggesting statistically significant changes, prompting a change in the centerline.

### Ethical Considerations

Per institutional policy, this project was not considered human subjects research and was deemed exempt by our local institutional review board quality improvement certification process.

## RESULTS

**Fig. 4.**
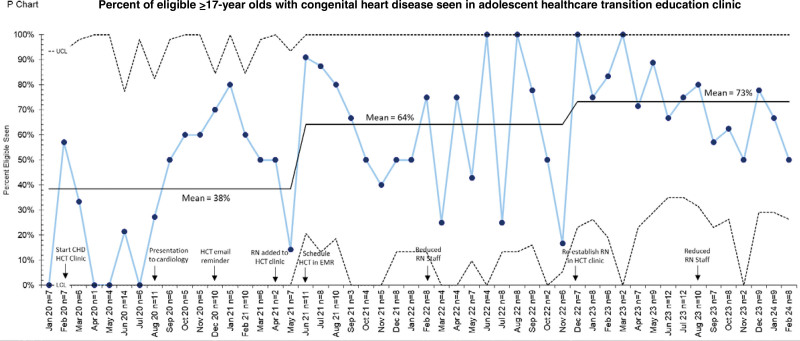
Statistical process control p-chart of percent eligible adolescents 17 years and older with congenital heart disease seen in the healthcare transition education clinic. LCL, lower control limit; RN, registered nurse; UCL, upper control limit.

A sustainable, structured HCT process has been developed since the CHD HCT clinic was implemented in February 2020. The control chart shows the percentage of eligible adolescents 17 years and older in the CHD HCT clinic (Fig. 4). Improvement was achieved in this group receiving HCT education, shifting the center line from 38% to 64% by June 2021 and another shift to 73% by December 2022. These shifts were temporally associated with incorporating nursing staff into the HCT clinic and scheduling HCT visits in the EMR. Although this did not quite reach the SMART goal of 75%, since December 2022, we have improved the wide range of percentage of HCT education from month to month and continue to work toward consistent educator presence to improve the success rate.

The Pareto chart (Fig. 3) indicates the etiology of missed HCT visits in those 17 and older from 2020 to 2024. Several missed opportunities for HCT visits continue, primarily due to staffing limitations of the HCT team. These include late add-on pediatric cardiology clinic visits not captured by our current system, staff unavailable to provide HCT education or an eligible patient missed during the initial scheduling of HCT visits. The re-establishment of nursing support to the HCT clinic from December 2022 to August 2023 has been associated with reduced missed visits secondary to unavailable staff and an improvement in the identification of eligibility. Communication was the largest factor of missed HCT education in 2021 (nine of 30 missed due to communication, 30%), for example, providers would forget, and the adolescent would leave before HCT education. Because adding scheduled HCT clinic visits in the EMR and provider reminders, the workflow has become embedded into the process, resulting in zero missed HCT education due to communication since 2022.

Comparing the survey results of pediatric cardiology providers from 2020 to 2023, cardiology providers had a greater agreement that we have a formal HCT process (3.8 versus 4.9, *P* = 0.02, ranging from one to five with a higher value representing more agreement), successful support for HCT clinic education (3.7 versus 4.8, *P* = 0.01). They successfully incorporated a structured HCT process into our workflow (2.9 versus 4.5, *P* = 0.004). An overall rating (1–10, with a higher value representing more agreement) that our current HCT process is meeting the needs of the providers, patients, and families increased from 5.5 to 7.8 (*P* = 0.01).

Demographics of the individual adolescents who completed a baseline TRA are shown in Table 1. Baseline TRA questionnaires were completed by 101 individuals (56% men, 44% women) with a mean (SD) age at the first survey of 17.9 years (1.9), age range from 12.1 to 22.8 years. A total of 60 (59%) had a cardiac procedure, with 50% of them having one procedure and the other 50% having two to six cardiac procedures with the median (IQR) age at first surgery requiring CPB 10.8 months (2.4–36 months). As documented in the medical record, 36 (36%) have a disability. Of the overall cohort, less than 65% of all respondents reported adequate knowledge in 10 of 18 healthcare knowledge questions and processes (Table 2).

**Table 1.** Demographics of Individual Patients who Completed a baseline Transition Readiness Assessment Questionnaire

	Study Population, N = 101
Sex, N (%) Male Female	57 (56) 44 (44)
Age at first survey, mean (SD), y Range, y	17.9 (1.9) 12.1–22.8
Cardiac surgery, N (%)	60 (59)
Total no. surgeries or cath, N (%) 0 1 2 3 4 5 6	41 (41) 30 (30) 8 (8) 12 (12) 6 (6) 2 (2) 2 (2)
Age of first surgery that required CPB, median (IQR), mo	10.8 (2.4–36)
Total no. surgeries that required CPB, N (%) 0 1 2 3	3 (5) 42 (70) 10 (17) 5 (8)
Have disability, N (%)	36 (36)

IQR, interquartile range.

**Table 2.** Transition Readiness Assessment Questionnaire Adolescent Responses by Cardiac Procedure versus No Procedure, Disability versus No Disability, and Overall Data for the Entire Group

	With a Cardiac Procedure (N = 60)	Without a Cardiac Procedure (N = 41)	*P*	With a Disability (N = 36)	Without a Disability (N = 65)	*P*	Overall (N = 101)
Confidence (range from 0 to 10, with a higher score representing more confident), mean (SD)							
Overall confidence in taking charge of heart health care	6.9 (2.9)	7.3 (2.3)	0.47	6.3 (3.5)	7.5 (2.1)	**0.04**	7.1 (2.7)
Overall confidence in moving to adult care	6.2 (3.2)	7.0 (2.4)	0.22	4.9 (3.5)	7.4 (2.1)	**<0.001**	6.5 (2.9)
My health, N (%)							
I can name my heart condition	31 (57)	27 (73)	0.13	16 (46)	42 (75)	**0.005**	58 (64)
I can describe the heart surgeries or procedures I have had	25 (51)	NA	NA	10 (50)	15 (52)	0.91	25 (51)
I know the name and doses of my medications*	33 (77)	23 (100)	**0.01**	21 (78)	35 (90)	0.3	56 (85)
I know my allergies to medications*	34 (77)	21 (91)	0.20	19 (70)	36 (90)	**0.05**	55 (82)
I know the name and contact of my cardiologist	29 (56)	20 (54)	1.00	15 (44)	34 (62)	0.10	49 (55)
I know I need lifelong heart care	44 (90)	25 (71)	**0.03**	25 (83)	44 (82)	0.83	69 (82)
I know I need to maintain health insurance	42 (81)	32 (87)	0.48	24 (71)	50 (91)	**0.01**	74 (83)
Using health care, N (%)							
I feel comfortable asking my provider questions	41 (77)	36 (97)	**0.01**	22 (65)	55 (98)	**<0.001**	77 (86)
I answer my providers questions on my own	31 (59)	26 (70)	0.28	14 (41)	43 (77)	**0.001**	57 (63)
Before a visit, I think about questions to ask	28 (56)	27 (75)	0.07	16 (53)	39 (70)	0.13	55 (64)
I know to ask my provider for recommendations if I need to see other doctors	34 (64)	29 (78)	0.15	16 (47)	47 (84)	**<0.001**	63 (70)
I take part in making choices about my health care	33 (70)	32 (89)	0.06	20 (69)	45 (83)	0.13	65 (78)
I know how to refill my medications*	23 (50)	16 (55)	0.66	14 (45)	25 (57)	0.32	39 (52)
I know what to do if I have a medical emergency	37 (71)	33 (89)	0.07	23 (68)	47 (86)	**0.05**	70 (79)
I know how to contact my health insurance company with questions or concerns	11 (23)	11 (31)	0.40	5 (17)	17 (31)	0.15	22 (26)
I have a paper or electronic file for my medical information	21 (41)	20 (54)	0.23	12 (35)	29 (54)	0.09	41 (47)
I understand how healthcare privacy changes for adults	26 (51)	19 (51)	0.97	16 (47)	29 (54)	0.54	45 (51)
I carry important health information with me every day	20 (39)	18 (49)	0.38	16 (47)	22 (41)	0.56	38 (43)

* Respondents were asked yes/no/not applicable, and not applicable was excluded from the denominator.

NA, not applicable; P, probability.

The baseline TRA results analysis showed that those with and without cardiac procedures were similarly confident in taking charge of their health care (6.9 versus 7.3, *P* = 0.47) and moving to adult care (6.2 versus 7, *P* = 0.22). Compared with those without a prior cardiac procedure, those with a cardiac procedure knew the name of their medications less often (77% versus 100%, *P* = 0.01) and felt comfortable asking a doctor or nurse questions less often (77% versus 97%, *P* = 0.01). A higher percentage of adolescents (90% versus 71%, *P* = 0.03) who had a cardiac procedure reported knowing they needed lifelong heart care compared with those who had no cardiac procedure. Adolescents with a disability were significantly less confident in taking charge of their heart health care than those without disabilities (6.3 versus 7.5, *P* = 0.04). This group was also less confident moving to adult care (4.9 versus 7.4, *P* < 0.001), and more variables indicated that those with disabilities had less knowledge about HCT topics compared with those adolescents without disabilities. Table 2 shows the results of the TRA confidence and healthcare knowledge for the overall cohort and the subgroups (procedure versus no procedure, disability versus no disability).

## DISCUSSION

The pediatric cardiology clinic successfully increased the consistent delivery of HCT education and the use of a TRA to adolescents with CHD. This increase has been sustained since June 2021, nearly achieving the initial SMART aim for those 17 years and older by December 2022. Consistent HCT educator and clinic nursing support was associated with reducing the monthly variability and achieving the highest consistent percentage of 17 years and older receiving HCT education. Notably, HCT knowledge and confidence were lowest for adolescents with disabilities.

Achieving successful HCT education poses challenges within the structure and function of a busy specialty clinic. Studies report clinical barriers include lack of clinician time, communication, care coordination support, and developmentally appropriate resources.^28,31,32^ Building processes with appropriate availability and flexibility of key HCT educators will likely improve success rates as the most common reasons for missed HCT education were a late clinic visit schedule, no staff availability, or a missed opportunity to schedule during the monthly review. In our clinic and HCT scheduling processes, we initially relied primarily on one HCT team member with limited coverage if that individual had other conflicting responsibilities. Incorporating nursing into the HCT team has impacted successful HCT education and is now included in the nursing routine. As seen in busy clinics with shifting roles and responsibilities,^8^ we need consistent nursing involvement in HCT education. This continues to be the biggest hurdle to overcome. Based on our experiences thus far, having at least two people with availability each day would provide coverage more consistently. We included nursing support for HCT education as the best fit for our clinic needs. However, each clinic could involve other team members to provide HCT education based on their clinic workflow. This supports the longer-term global aim to expand eligibility criteria and increase the number of adolescents who receive HCT education. With the goal of expanding to include more youth between 12 and 16 years of age, PDSA cycles will continue to monitor for impact on factors that limit the HCT clinic education. Additionally, future considerations to offer HCT education via telehealth could provide flexibility in scheduling, may allow added success when an in-person HCT scheduling conflict arises, and expand the inclusion to additional adolescents from clinic sites more distant from the primary site.

An important finding of this study is that adolescents with disabilities had lower knowledge and confidence in HCT. Although evidence for the importance of HCT in adolescents with chronic conditions is extensive,^27,28,33,34^ few studies investigate differences in HCT preparation for adolescents with and without disabilities.^35,36^ It has been reported that receiving special education was negatively related to receiving HCT support.^27^ Although the data in this study suggest that HCT knowledge gaps exist for all adolescents, it is important to highlight the findings that adolescents with CHD and disabilities may have different needs relating to their knowledge and confidence in HCT readiness to improve their engagement and long term health; these are important areas for future research.

This study’s limitations include the fact that the results of our HCT clinic may need to be more generalizable to other institutions as the study occurred in a single institution. Depending on the reliability and validity of documentation, the use of EMR data in determining those with and without disabilities may be incomplete.

The data from this study show that adolescents with CHD have moderately high levels of confidence and lower levels of knowledge regarding HCT. Those with disabilities are less confident and knowledgeable compared with those without disabilities, reflecting an important disparity. The etiology of these differences should be studied, and methods to improve HCT should be identified. Many adolescents with CHD have difficulty identifying their heart condition, struggle with healthcare insurance or medications, and lack coordinated education support. Strategies to improve the quality of HCT should be useable by the greatest number of adolescents, including those with disabilities. Current interventions often focus on individual patient knowledge. However, many patient outcome measures do not evaluate quality indicators such as developmental, vocational, or psychologic aspects of HCT and additional research focusing on adolescent HCT as it relates to their specific social, family, and community environments could advance improvements in HCT.^28,37^ In a time of significant medical and surgical advances with many successes in the healthcare field, where more children and adolescents survive to become adults than past generations, ongoing research and investment into the future of these patients is paramount. Improving the HCT education and process, together with research inclusive of diverse adolescents, families, care providers, and community partners, is key to helping adolescents become strong advocates for their health, minimize being lost in the transition to adult health care, and thrive well into adulthood.
